# LipidCruncher: An open-source web application for processing, visualizing, and analyzing lipidomic data

**DOI:** 10.1101/2025.04.28.650893

**Published:** 2025-05-01

**Authors:** Abdi Hamed, Yohannes A. Ambaw, Chandramohan Chitraju, Shubham Singh, Zon Weng Lai, Robert V. Farese, Tobias C. Walther

**Affiliations:** 1 Cell Biology Program, Sloan Kettering Institute, New York, NY, USA.; 2 mRNA Center of Excellence, Sanofi, Waltham, MA, USA.; 3 Howard Hughes Medical Institute, New York, NY, USA.

**Keywords:** lipidomics, data analysis, quality control, standardization, anomaly detection, visualization, volcano plots, saturation profiles, lipid pathways, lipidomic heatmap

## Abstract

Advances in mass spectrometry (MS)-based lipidomics have led to a surge in data volume, underscoring a need for robust tools to evaluate and visualize these data comprehensively. Current workflows are often hampered by manual spreadsheet handling and insufficient assessment of data quality prior to analysis. Here, we introduce *LipidCruncher*, an open-source, web-based platform designed to process, visualize, and analyze lipidomic data with high efficiency and rigor. *LipidCruncher* consolidates key steps of the workflow, including data standardization, normalization, and stringent quality control to identify anomalies. The platform also provides advanced visualization and analysis tools, such as volcano plots, lipid saturation profiles, pathway mapping, and lipid heatmaps, that enable detailed and holistic data exploration. To demonstrate *LipidCruncher*’s utility, we analyzed lipidomic data from adipose tissue of mice lacking the triacylglycerol synthesis enzymes DGAT1 and DGAT2. We anticipate that *LipidCruncher* will be a valuable and user-friendly tool for standardizing and analyzing lipidomics data.

## INTRODUCTION

Lipids include diverse classes and thousands of lipid species [1, 2], presenting challenges in identifying changes and their patterns under different physiological conditions. Despite these complexities, precise quantification and characterization of lipid species are essential for dissecting the intricate web of metabolic and signaling pathways involving lipids.

The emergence of liquid chromatography coupled to mass spectrometry (LC-MS/MS) and “shotgun” approaches have revolutionized the field of lipidomics and enabled the detection of a large (hundreds to thousands) and diverse array of lipid species with unprecedented depth and breadth [3–5]. A current challenge lies not in the generation of data but in its analysis and interpretation.

Lipidomic data analysis involves several key steps. First, mass spectra are processed using specialized software tools (e.g., *LipidSearch*, *LipidXplorer* [6], LipidFinder [7] and *MS-DIAL* [8]) that assign specific lipid species to ion current peaks at particular mass-to charge ratios and quantify their abundance, while utilizing fragmentation spectra of corresponding ions to enhance identification accuracy and structural characterization. These tools turn the mass spectra into a structured dataset input for subsequent bioinformatics analysis.

Other software tools (e.g., *lipidr* [9], *LipidSig* [10], and *ADViSELipidomics* [11]) bridge the gap from structured lipidomic datasets to biological insights by providing sophisticated features for processing, visualizing, and analyzing lipidomic data. However, significant gaps in this process remain. Many of these tools require complex installation, lack user-friendly interfaces, or fail to support diverse data input formats. Moreover, comprehensive workflows that integrate robust data normalization and quality assessment are often lacking. These limitations underscore the need for a streamlined, intuitive platform that provides users an end-to-end solution for analyzing lipidomics data.

To address these challenges and meet the expansive needs of lipidomics research, we developed *LipidCruncher*. This open-source, web-based tool accommodates diverse data formats and streamlines the transition from semi-processed data to outputs that allow for scientific insights. It integrates a robust framework for data standardization and normalization with rigorous quality checks to ensure the integrity and reliability of the analysis. *LipidCruncher*’s suite of interactive visualizations provides an intuitive interface that bridges the gap between complex lipidomics data and biological interpretation. *LipidCruncher* simplifies and standardizes the workflow, enabling users to systematically analyze lipidomics data with consistency and confidence.

## RESULTS

### Description of *LipidCruncher* workflow

#### Module 1: Data input, standardization, filtering, and normalization

**Figure 1.**
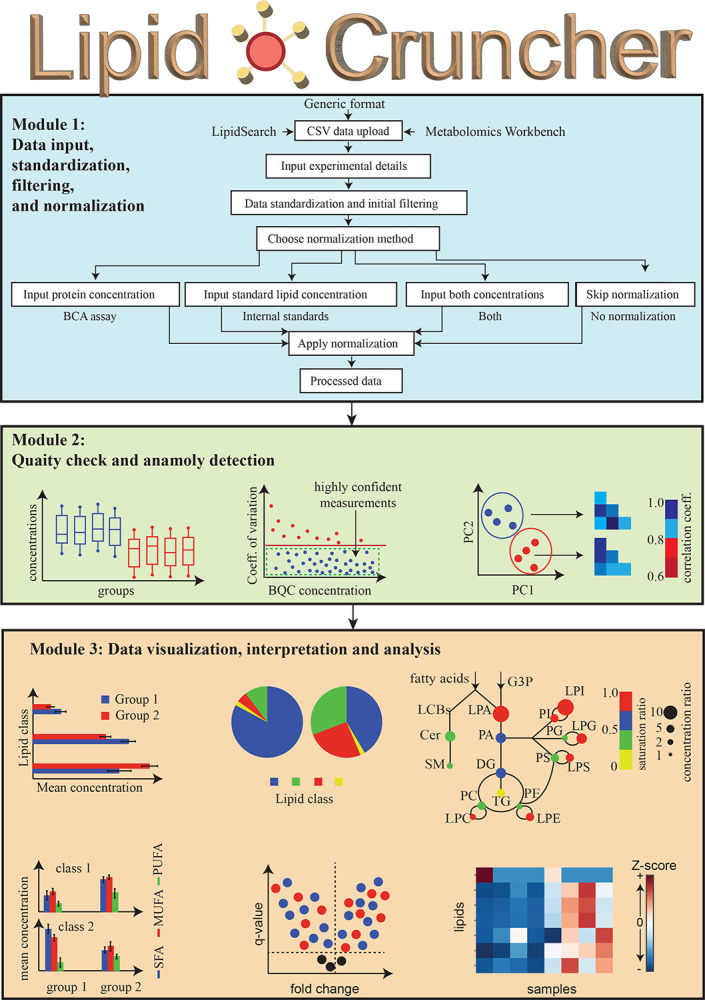
Overview of the *LipidCruncher* analysis pipeline. The *LipidCruncher* workflow is organized into three modules. Module 1 handles standardization, initial filtering, and normalization of the inputted data. Module 2 verifies data uniformity across replicates using box plots, crucial for reliable comparison, and evaluates technical replicate consistency through batch quality control (BQC) assessments to ensure data fidelity. Anomaly detection employs principal component analysis (PCA) for dimensional reduction and visual outlier identification, complemented by correlation heatmaps to enhance anomaly detection through biological replicate comparison. Module 3 features a suite of visualization tools, starting with concentration bar and pie charts for quantitative and proportional data insights. Metabolic network contexts and saturation profiles offer deeper dives into the lipidome structure, while the interactive volcano plot and comprehensive lipidomic heatmap with z-score color coding and clustering highlight significant lipidomic alterations and complex data relationships.

The workflow and features of *LipidCruncher* include modules that group three stages of analysis (Figure 1). The first step of Module 1 is data input, for which *LipidCruncher* is designed to accommodate outputs from various software platforms. Users can provide data in a generic format (Supplementary File 1) or import datasets from *LipidSearch* (Supplementary File 2) or *Metabolomics Workbench* (Supplementary File 3) [12]. Inputs must be provided as a CSV file.

For a generic format, input data are organized with a dedicated column for lipid species classification and structure, accompanied by multiple columns containing values that represent the abundance of each lipid species in a particular study sample. The values are usually derived from the area under the curve of each lipid species’ peak in the mass spectrum, reflecting their relative abundances. For example, in a study with 12 biological samples—four replicates of three biological conditions—the dataset would consist of rows corresponding to distinct lipid species (e.g., PC (16:0_18:2)). The first column lists unique lipid identifiers followed by twelve columns each containing the intensity/abundance values for this lipid detected in each sample (Supplementary File 1).

Input files from *LipidSearch* or *Metabolomics Workbench* contain additional data columns beyond lipid names and intensities. For instance, *LipidSearch* files include calculated mass, retention time, quality grades, and other lipid-specific metrics, and *Metabolomics Workbench* files include sample information and experimental conditions in a structured format. These additional columns enable *LipidCruncher* to perform specialized analyses that are not possible with the generic input option.

For optimal experimental design and reliable results in lipidomics studies, we recommend several best practices. First, we recommend having at least 4–6 biological replicates for each condition to ensure statistical rigor and account for biological variability. Second, the creation of at least three batch quality control (BQC) samples is crucial. BQC samples are technical replicates generated by pooling equal aliquots from each experimental sample and are crucial for evaluating measurement consistency and monitoring instrument performance throughout the analysis. Lastly, to ensure robust data analysis, we advise using both internal standards (spiked in lipid molecules of known concentration) and protein-based normalization methods.

The process of data standardization and normalization is outlined as Module 1 of Figure 1. Upon data upload, *LipidCruncher* automatically initiates a standardization and normalization protocol. To begin, the user is prompted to enter essential experimental parameters, including the number of conditions, descriptive labels for each condition (such as ‘WT’ for wild type), and the count of replicates per condition. Additionally, the user is asked if the data include BQC samples.

During data standardization, *LipidCruncher* ensures uniform column naming to streamline downstream analyses. The column containing lipid species information, including class and structural details, is standardized as “LipidMolec.” Intensity data for each sample are formatted as “intensity[S_1_],” “intensity[S_2_],” …, “intensity[S_N_],” where ‘N’ corresponds to the total number of samples in the experiment. This standardized structure provides consistency, enabling seamless integration with the subsequent normalization steps.

*LipidCruncher* next performs a data-cleaning step to ensure the dataset is prepared for analysis. This involves removing empty rows and duplicate entries, as well as replacing ‘null’ values with zeros to preserve computational integrity. While *LipidCruncher* generally does not impute missing values, it does implement a targeted imputation strategy specifically for log transformation during statistical significance testing. For other analyses, it interprets missing values as analytes likely below the detection threshold of the mass spectrometer, representing negligible abundance.

The final step in the preparatory workflow is normalization. *LipidCruncher* provides four normalization options: (1) no normalization for datasets already normalized externally, (2) normalization using internal standards, (3) normalization to protein amount, or (4) normalization using both internal standards and protein measurements.

For internal standard based normalization (options 2 and 4), users include a mix of labeled standards spiked into the samples prior to lipid extraction to ensure precise measurements. *LipidCruncher* automatically identifies and segregates the internal standards of the SPLASH LIPIDOMIX^®^ Mass Spec Standard (Avanti Polar Lipids, Cat# 330707–1), based on their nomenclature [e.g., DG (15:0_18:1) +D7:(s) is recognized as a standard for the DG class] (Supplementary File 4). If users employ alternative standards with different naming conventions, they are prompted to upload a list specifying the standards in their samples. Before normalization, *LipidCruncher* organizes the internal standards, allowing users to assess the uniformity of standard abundance across samples. The platform then prompts users to assign the appropriate internal standard to each lipid class and input its concentration. Using the known quantities of these standards, *LipidCruncher* calculates the absolute concentrations of lipid species and transforms relative abundance data into normalized concentration values.

For protein-based normalization (options 3 and 4), users must independently determine the concentration of proteins in their samples (e.g., using a bicinchoninic acid assay). Protein measurements act as a proxy for the total biological material from which lipids were extracted, providing a normalization factor based on sample protein content.

After the normalization process is complete, the column names are updated to reflect the transformed data. The intensity columns (“intensity[S_1_],” “intensity[S_2_],” …, “intensity [S_N_]”) are renamed to “concentration[S_1_],” “concentration[S_2_],” …, “concentration [S_N_]” to indicate that the values now represent normalized concentrations rather than raw intensities.

#### Module 2: Quality check and anomaly detection

After normalization, *LipidCruncher* transitions to the “quality check and anomaly detection” phase, a critical step for ensuring data integrity and quality. As an initial diagnostic, the platform generates a series of box plots for each sample, displaying the concentration distributions of all detected analytes with nonzero values (Module 2 of Figure 1). These plots allow for rapid assessment of data quality, with replicates from the same experimental condition expected to exhibit similar medians, interquartile ranges, and overall distributions. Such uniformity across replicates is indicative of consistent sample preparation and data acquisition.

In addition to box plots, a bar chart quantifies the percentage of zero values per sample (not shown in Fig. 1). This chart helps identify anomalies, such as samples with an unusually high proportion of zero values or significant deviations in distribution patterns among replicates.

*LipidCruncher* further evaluates data quality by calculating the coefficient of variation (CoV) for BQC samples (Module 2 of Figure 1). The software filters out data with a CoV larger than 30%, a threshold that can be adjusted by users, based on their specific needs and experimental requirements. A relatively low CoV indicates high fidelity of the data, reflecting reproducible and reliable measurements throughout the experimental workflow.

After CoV calculation, users are given an option to remove lipid species with CoV values above their chosen threshold. For more selective filtering, users can also remove individual species by specifying their row indices, providing flexibility when they prefer not to apply a blanket removal of all species above the threshold. We recommend running the complete analysis pipeline both with and without the species marked for deletion to evaluate their impact on experimental outcomes. This comparative approach ensures that the filtering decisions are scientifically sound and do not inadvertently affect the study’s conclusions.

The integrity of the dataset is further evaluated using pairwise correlation and principal component analysis (PCA), [13] as shown in Module 2 of Figure 1. For correlation analysis, zero values are excluded, and a heatmap of correlation coefficients is generated for all replicates. Biological replicates typically exhibit correlation coefficients greater than 0.7, indicative of high reproducibility. Deviations from this threshold may signal potential outliers, arising either from biological variability or procedural inconsistencies.

PCA, in contrast, incorporates zero values to reduce dataset dimensionality and distills it into principal components that capture the greatest variance. The resulting biplot of the first two principal components (PC1 and PC2) provides an intuitive visual representation of replicate clustering. Confidence ellipses (95% confidence interval) are drawn around each experimental condition to facilitate the interpretation of group separation. Samples falling outside these confidence intervals are flagged as potential outliers, suggesting anomalies in sample preparation, data acquisition, or inherent biological differences.

When anomalies are identified, *LipidCruncher* provides users with the option to exclude aberrant samples from the dataset. If an anomaly is determined to stem from experimental errors, its removal is recommended to maintain the integrity of the analytical results.

#### Module 3: Data visualization, interpretation, and analysis

The final phase of the *LipidCruncher* pipeline focuses on comprehensive analysis and visualization of lipidomic data, as summarized in Module 3 of Figure 1. All visualizations generated by *LipidCruncher* are interactive, promoting user engagement and facilitating detailed data exploration. These interactive features allow users to investigate specific data points by hovering a mouse cursor over them, revealing detailed information, such as lipid names and their corresponding measurement values.

The platform begins by generating bar plots (Module 3 of Figure 1) that display the mean concentrations of lipid classes across experimental conditions. Error bars denote the standard deviation of replicate measurements, and statistical significance between conditions is evaluated with appropriate tests. To compare two conditions, Welch’s t-test is applied [14], whereas an ANOVA with Tukey’s post-hoc test is used for multiple conditions. To account for multiple comparisons, p-values are adjusted using the Benjamini-Hochberg procedure [15]. Bar plots are provided on both linear and logarithmic scales, enabling detailed and context-appropriate interpretation.

To complement the bar plots, *LipidCruncher* also generates pie charts that illustrate the proportional concentrations of lipid classes across conditions (Module 3 of Figure 1). These visualizations provide an intuitive representation of lipid class distributions, offering an immediate grasp of compositional differences.

In addition to visualizing individual lipid classes and species, identifying patterns of change across lipid analytes provides valuable insights into metabolic alterations. To facilitate this, *LipidCruncher* includes a visualization of lipid classes within the context of a metabolic network, focusing on major lipid classes (Module 3 of Figure 1). In this network, each lipid class is represented by a color-coded circle. The diameter of the circle corresponds to the concentration ratio of that class between two selected conditions. The intensity of the circle’s color reflects the saturation ratio, defined as the ratio of saturated fatty acids to the total fatty acid content.

*LipidCruncher* also performs a detailed fatty acid saturation profile analysis, as illustrated in Module 3 of Figure 1. This analysis breaks down each lipid class into its constituent fatty acid types: saturated fatty acids (SFA), mono-unsaturated fatty acids (MUFA), and poly-unsaturated fatty acids (PUFA). The absolute concentrations of these fatty acid categories are presented in bar plots, which include standard deviation error bars and indicators of statistical significance. For two-condition comparisons, Welch’s t-test is used, whereas multiple conditions are analyzed with ANOVA and the Tukey’s post-hoc test. P-values are adjusted for multiple comparisons using the Benjamini-Hochberg procedure to ensure robustness. In addition to the absolute data, a complementary stacked bar chart depicts the relative percentage distribution of each fatty acid type within each lipid class.

*LipidCruncher* further enhances data interpretation by generating volcano plots (Module 3 of Figure 1), which plot the fold-change of each lipid analyte against the statistical significance of its change. The analysis employs Welch’s t-test and p-values are adjusted for multiple comparisons using the Benjamini-Hochberg method. This format efficiently identifies significant lipidomic changes, with analytes exhibiting substantial alterations located in the upper-outer quadrants.

To tailor analysis to specific needs, users can customize several parameters: (1) selecting control and experimental conditions from datasets with multiple replicates, (2) adjusting the significance threshold (default p-value = 0.05), (3) filtering specific lipid classes for visualization, and (4) optionally hiding non-significant data points to declutter the display. Additionally, a complementary plot (not shown in Figure 1) visualizes the fold-change of each lipid analyte against its mean concentration in control samples, offering insights into the relationship between a lipid’s abundance and its degree of change.

For more granular exploration, *LipidCruncher* generates concentration distribution plots for selected lipids across experimental conditions. These are presented as box plots (not shown in Figure 1), enabling users to assess variability and trends in individual lipid levels.

Finally, *LipidCruncher* offers the option to visualize lipidomic data as heatmaps (Module 3 of Figure 1), where rows correspond to individual lipids and columns represent samples. *LipidCruncher* provides two options for heatmaps. The first preserves the original order of the lipids, and the second applies Ward’s hierarchical clustering method to the lipid concentration data, grouping lipids with similar concentration patterns across samples into clusters [16]. Lipids within the same cluster typically share comparable expression profiles across experimental conditions, suggesting potential biological relationships. Heatmaps allow users to discern subtle trends and groupings that might otherwise remain undetected. The heatmaps utilize z-score color coding to enable standardized comparisons across the dataset.

For data export, *LipidCruncher* provides versatile options, catering to both in-depth analysis and presentation needs. Users can download plot data in CSV format for offline examination or export visualizations as SVG files, allowing for further customization and seamless integration into reports or presentations.

To streamline documentation, *LipidCruncher* generates a comprehensive PDF report summarizing the entire analysis. This report includes all key findings, visualizations, and statistical results, and can be downloaded with a single click. By simplifying the compilation and sharing of analytical outputs, this feature enhances the efficiency of lipidomic research workflows, supporting both data exploration and communication of results.

### Case study utilizing *LipidCruncher* to analyze a lipidomic dataset

#### Data input and data quality analysis for the case study

**Figure 2.**
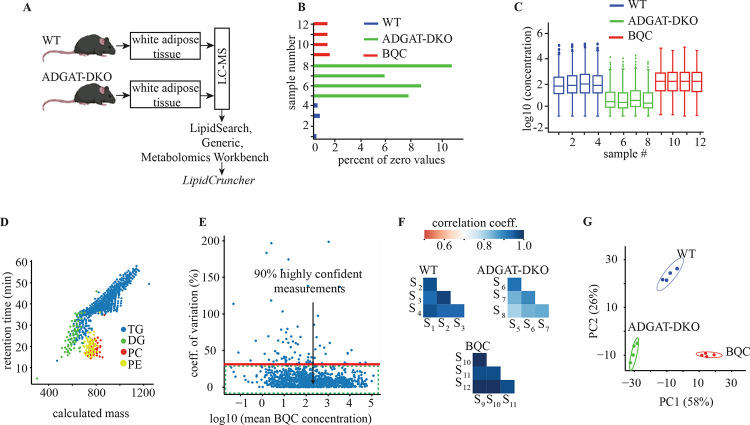
Comprehensive quality check analysis of lipidomic dataset from DGAT1/DGAT2 double knock out (ADGAT-DKO) mice. A) The experimental design is displayed. B) Bar plot showing the percentage of missing (zero) values in WT and ADGAT-DKO samples, revealing a higher proportion in ADGAT-DKO (~8%) than WT (~0.4%), suggesting potential lipidomic alterations due to DGAT enzyme knockout. C) Box plots displaying the distribution of normalized lipid concentrations across WT, ADGAT-DKO, and BQC samples. The uniform median and interquartile range distributions within each condition validate the normalization process and indicate dataset reliability. D) Scatter plot of retention time versus calculated mass for representative lipid classes (TG, DG, PC, and PE) demonstrates the expected clustering patterns based on hydrophobicity and mass. This plot serves as a crucial quality check for lipid identification consistency. E) Scatter plot of Coefficient of Variation (CoV) against mean concentration: 90.0% of lipid species exhibit a CoV less than 30%, indicating high measurement precision. F) Heatmaps displaying correlation coefficients between replicates within WT and ADGAT-DKO conditions. High correlation coefficients (>0.8) demonstrate strong replicative consistency within each experimental group. G) PCA plot showing distinct clustering of WT and ADGAT-DKO samples, underscoring significant lipid composition differences induced by the genetic modifications.

To demonstrate the workflow and utility of *LipidCruncher*, we present a case study using lipidomic data from murine adipose tissue, where the enzymes catalyzing triglyceride synthesis, DGAT1 and DGAT2, were deleted (ADGAT-DKO mice) [17] (Figure 2A). The study included two primary experimental groups: WT and ADGAT-DKO mice, each with four biological replicates. Additionally, four BQC samples were included to assess analytical precision. After sample extraction, lipid analysis was performed using LC-MS/MS. Raw data were initially processed using *LipidSearch* 5.0, and resultant CSV output files served as the input for *LipidCruncher* [Supplementary Files 2 and 4].

*LipidCruncher* processed the input dataset, assigning sample identifiers *S*_1_ to *S*_12_ across the WT, ADGAT-DKO, and BQC groups. After data standardization and filtering, *LipidCruncher* automatically generated a refined dataset containing 1042 endogenous lipid species spanning 17 lipid classes (AcCa, BMP, Cer, ChE, CL, Co, DG, HexCer, LPC, LPE, PC, PE, PG, PI, PS, SM, TG), along with nine internal standards: DG(15:0–18:1)+D7(s), LPC(18:1)+D7(s), LPE(18:1)+D7(s), PC(15:0–18:1)+D7(s), PE(15:0–18:1)+D7(s), PG(15:0–18:1)+D7(s), SM(d36:2)+D9(s), ChE(18:1)+D7(s), and TG(15:0–15:0–18:1)+D7(s). To ensure comparability across lipid classes, *LipidCruncher* performed normalization using internal standards with the closest structural similarity to each lipid class [Supplementary File 4].

An analysis of the dataset (Figure 2B) revealed a higher average proportion of missing (zero) values in the ADGAT-DKO samples (~8%) than in the WT samples (~0.4%), indicating substantial lipidomic changes due to the deletion of the genes encoding the DGAT enzymes. This observation is supported by the low variation between replicates within each condition, as shown by the uniform median and interquartile range distributions of total lipid concentration in Figure 2C. This consistency between replicates is a sign of high-quality data and validates the normalization process, particularly evident in the technical BQC replicates. Any substantial deviation in these BQC samples would have indicated technical issues in the experimental workflow, necessitating thorough examination and troubleshooting.

Figure 2D illustrates a plot of retention time versus calculated mass for lipid species, providing an essential quality check for the dataset. This analysis is based on the principle that a lipid’s retention time in a reverse-phase chromatography column correlates with its hydrophobicity. Retention time—the duration a molecule takes to traverse the column—increases for more hydrophobic molecules due to their stronger interactions with the hydrophobic stationary phase. This relationship produces a characteristic pattern in the plot, which serves as a key tool for evaluating the consistency and accuracy of lipid identification.

To illustrate key patterns in the data, Figure 2D highlights four representative lipid classes: TG, DG, PC, and PE. While all lipid classes are visible in *LipidCruncher*, these four were selected for the figure to demonstrate the distinct clustering behavior that reflects their distinct physical and chemical properties. Among these highlighted classes, TGs, the most hydrophobic lipids, display the longest retention times. The clustering patterns observed in the plot align with the expected behavior of these lipid classes, further supporting the dataset’s reliability. Any outliers or deviations from the expected cluster positions could indicate potential misidentifications or experimental anomalies and would necessitate further investigation.

Further confidence in the lipid measurements was derived from the analysis of the CoV for each lipid (Figure 2E), which showed that the majority (>90.0%) of lipid species had a CoV below 30% across BQC samples. We opted to remove the lipid species with higher than 30% CoV, indicating highly variable and possibly unreliable values, from subsequent analyses. However, this threshold is optional, and the choice of if/how to apply this filtering step is up to the researcher based on the specific nature of their experiment and research question. Additionally, strong correlations (correlation coefficients >0.8) between replicates in both WT and ADGAT-DKO conditions (Figure 2F) further validate the dataset’s reliability.

PCA plots (Figure 2G) provided additional evidence of the dataset’s robustness, with all samples clustering distinctly within their respective 95%-confidence ellipses. The difference between clusters highlights significant differences in lipid composition resulting from the genetic modifications. Importantly, no anomalies were detected during the analysis, confirming a reliable dataset with high replicative consistency.

#### Data visualization, interpretation, and analysis for the case study

**Figure 3.**
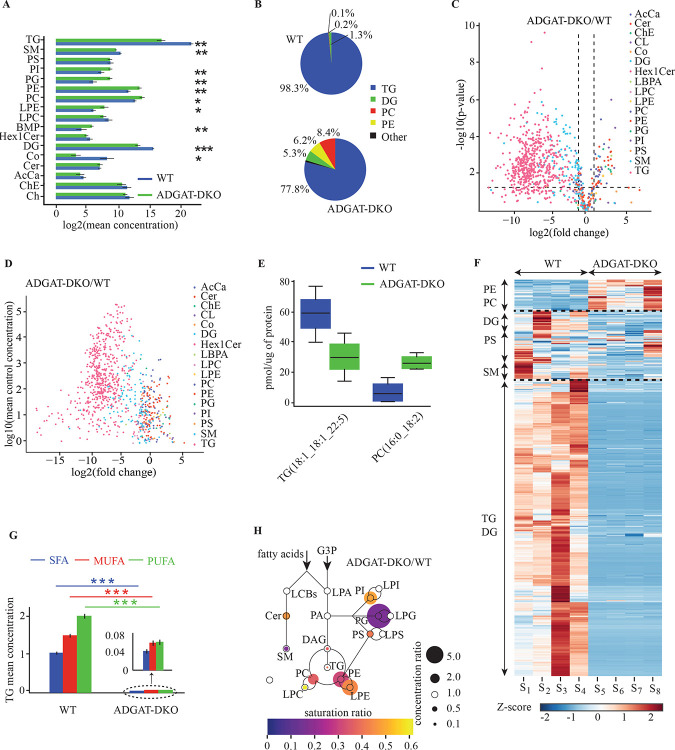
Comprehensive differential lipid analysis of DGAT1/DGAT2 double knock out (ADGAT-DKO) mice. A) Bar graphs comparing mean lipid class concentrations in WT and ADGAT-DKO mice, highlighting the substantial impact of DGAT1 and DGAT2 gene deletions. Notable is the 96.6% reduction in triglyceride (TG) levels in ADGAT-DKO mice. Statistical significance is indicated by asterisks, where * denotes p<0.05, ** denotes p<0.01 and *** denotes p<0.001. B) Pie charts illustrating the proportion of lipid classes in WT and ADGAT-DKO conditions. Despite significant reduction, TGs still represent a majority of the lipidome in ADGAT-DKO mice (decreasing from 98.3% to 77.8%), indicating potential metabolic robustness. C) Volcano plot providing a species-level comparison between WT and ADGAT-DKO conditions. This plot illustrates both the magnitude of change (fold change) and statistical significance for each lipid species, facilitating identification of biologically relevant alterations. D) Scatter plot of mean control concentration versus fold change, allowing examination of the relationship between a lipid’s baseline abundance and its degree of change between conditions. This visualization helps identify patterns across different lipid classes or concentration ranges. E) Box plot comparisons of two example lipids from the TG and PC classes across WT and ADGAT-DKO conditions. These plots visualize concentration distributions, including spread and potential outliers, providing a granular view of how specific lipid species are affected by the genetic modification. F) Lipidomic heatmap offering a high-resolution overview of lipidomic alterations between WT and ADGAT-DKO samples. The heatmap displays z-score-colored visuals for over a thousand lipid species, with an interactive hover-over feature for detailed information. Clustering rearranges the original lipid order, revealing patterns and associations not apparent in unordered data. G) Saturation profile analysis for TG species, revealing a shift from PUFAs towards monounsaturated fatty acids (MUFAs) in ADGAT-DKO samples, suggesting alterations in fatty acid metabolism and utilization. Statistical significance is indicated by asterisks, where * denotes p<0.05, ** denotes p<0.01 and *** denotes p<0.001. H) Lipidomic pathway visualization depicting dynamic changes in lipid classes between WT and ADGAT-DKO conditions. This diagram offers insights into the complex interplay between lipid biosynthesis and degradation pathways in the absence of DGAT enzymes.

The bar and pie charts in Figure 3A and 3B illustrate the substantial impact of DGAT1 and DGAT2 gene deletions on the lipidome. TG levels in adipose tissue of ADGAT-DKO mice are reduced by ~96%. Despite this dramatic reduction in total TG concentration, TGs remain the predominant lipid class, decreasing from 98.3% to 77.8% of the total lipid composition. These shifts highlight a significant reshaping of lipid concentrations and underscore a redirection of lipid metabolism in response to the loss of DGAT1 and DGAT2 activities. Notably, the depletion of TGs is accompanied by an **increase in amounts of phospholipids**, including **PC, PE, LPE**, and **BMP**. Conversely, amounts of **DG and SM were reduced**. These alterations indicate a metabolic adaptation, likely reflecting compensatory shifts in lipid synthesis and storage pathways.

The volcano plot (Figure 3C) offers a detailed lipid species–level comparison between WT and ADGAT-DKO samples, enabling the identification of specific lipids driving the observed phenotypic differences. This plot visualizes magnitudes of changes (fold-change) alongside the statistical significance of these changes, providing critical insights into the lipid species most impacted by the genetic modifications. The predominant finding is that multiple TG species are significantly reduced, and phospholipid species were elevated. Figure 3D presents a scatter plot illustrating the relationship between mean WT concentration and fold-change, with different lipid classes represented by distinct colors. The plot reveals a general trend of reduced TG and DG levels across both high- and low-abundance TG species, accompanied by an increase in levels of specific phospholipids. By examining these values, scientists can readily determine if highly abundant lipids are prone to significant changes and uncover patterns across different lipid classes or concentration ranges.

Figure 3E provides an example for examining specific lipids using box plot comparisons. The figure highlights the concentration distributions of representative lipid species from the PC and TG class across WT and ADGAT-DKO conditions. Such box plots allow researchers to visualize the average concentrations and the variability and potential outliers within each condition. This detailed perspective is essential for understanding the consistency of changes in lipid species and the impact of genetic modifications.

The lipidomic heatmap (Figure 3F) is a cornerstone of *LipidCruncher*’s capabilities, providing a high-resolution visualization of the complex lipidomic alterations between WT and DKO samples. With over a thousand lipid species, the data are organized into three distinct clusters for clarity. The upper cluster predominantly features PE and PC lipids, the middle cluster is enriched with DG, PS and SM species, and the bottom cluster primarily comprises TG and DG lipids.

The saturation profile analysis of TG species (Figure 3G) reveals a shift from PUFAs to MUFAs in the ADGAT-DKO samples, suggesting alterations in fatty acid metabolism and utilization. Additionally, the lipidomic pathway visualization in Figure 3H captures dynamic changes in lipid class concentrations, illustrating both increases and decreases while providing insights into the intricate interplay between lipid biosynthesis and degradation pathways in the absence of DGAT enzymes. The size of the circles indicates the relative concentrations of each lipid class, and the colors represent the saturation ratio.

Users can interact with the heatmap by hovering over each square in the map to view detailed information, including lipid identity and z-score. Additionally, the clustering process reorganizes the original lipid order, revealing patterns and associations that might otherwise go unnoticed in unordered data. This reorganization enhances the interpretation of lipidomic changes and provides insights into their potential biological significance.

## DISCUSSION

Here we present *LipidCruncher*, an open-source platform that simplifies and harmonizes lipidomic data analysis by integrating quality assessment, visualization, and statistical tools into a seamless workflow. Designed to accommodate diverse mass spectrometry outputs, it enables researchers to bypass technical bottlenecks and focus on extracting biological meaning from complex datasets. Built-in quality control safeguards ensure data integrity, minimizing errors and enhancing reproducibility. By streamlining analysis and fostering collaboration, *LipidCruncher* accelerates discovery and lays the foundation for a shared lipidomics data ecosystem, driving the field forward with rigor, transparency, and efficiency.

## MATERIALS AND METHODS

### Chemicals

The following reagents were purchased from commercial vendors. Acetonitrile, methanol, water (all HPLC/MS grade), chloroform (HPLC grade,), ammonium formate, ammonium acetate, formic acid, and acetic acid were from Sigma-Aldrich. SPLASH^®^ LIPIDOMIX^®^ Mass Spec Standard was from Avanti Polar Lipids, Cat# 330707–1EA.

### Mouse husbandry

Mice lacking triacylglycerol synthesis enzymes DGAT1 and DGAT2 in adipose tissue (ADGAT-DKO mice) were generated as described [17]. The previous report described selected lipidomic results rather than the analysis of the complete dataset. Mice were housed as per the guidelines from Harvard Center for Comparative Medicine. Mice were maintained in a barrier facility at room temperature (22°C) on a regular 12-h light and 12-h dark cycle. Mice had *ad libitum* access to food and water. Mice were fed on standard laboratory chow diet (PicoLab^®^ Rodent Diet 20, 5053; less than 4.5% crude fat). Inguinal white adipose tissue of 12-week-old male mice was used for lipid profiling.

### Sample extraction and LC-MS/MS analysis

Lipidomic profiling of inguinal white adipose tissue (iWAT) followed established protocols. Approximately 50 mg of iWAT was homogenized in 1 mL of ice-cold phosphate-buffered saline (PBS) using a bead mill homogenizer. Tissue lysates (50 μg) were then transferred to Pyrex glass tubes with polytetrafluoroethylene-lined caps for lipid extraction.

Lipids were extracted using the Folch method [18]. Briefly, 6 mL of ice-cold chloroform:methanol (2:1, v:v) was added to each sample, followed by 1.5 mL of water. To ensure thorough mixing of polar and non-polar phases, the tubes were vortexed vigorously. SPLAH mix internal standards were spiked into each sample before extraction. Protein concentrations were quantified using a bicinchoninic acid assay (Thermo Scientific, 23225, Waltham, MA) to normalize lipid amounts. After vortexing, samples were centrifuged at 1100 rpm for 30 min at 4°C to facilitate phase separation. The lower organic phase, containing extracted lipids, was carefully transferred to a new glass tube with a sterile glass pipette to ensure minimal disruption of the interphase layer containing cellular debris and precipitated proteins. Solvents were evaporated under a gentle nitrogen stream until complete dryness. The dried lipid extracts were reconstituted in 250 μL of chloroform:methanol (2:1, v:v) and stored at −80°C until further analysis.

For lipid separation and identification, ultra-high-performance liquid chromatography was coupled with tandem mass spectrometry (MS/MS). Lipid extracts were analyzed using a Thermo Acclaim C30 reverse-phase column (2.1 × 250 mm, 3 μm, Thermo Fisher Scientific) maintained at 55°C. The chromatographic system consisted of a Dionex UltiMate 3000 HPLC system coupled to a Q Exactive Orbitrap mass spectrometer (Thermo Fisher Scientific) equipped with a heated electrospray ionization (HESI) probe. Each sample (5 μL) was analyzed separately under both positive and negative ionization modes. The mobile phase composition was as follows: Mobile phase A: 60:40 (v/v) water:acetonitrile with 10 mM ammonium formate and 0.1% formic acid. Mobile phase B: 90:10 (v/v) 2-propanol:acetonitrile with 10 mM ammonium formate and 0.1% formic acid.

Mass spectrometric analysis was performed in full-scan/data-dependent MS^2^ mode. Full-scan spectra were acquired at a resolution of 70,000 with an automatic gain control target of 1 × 10^6^ and a maximum injection time of 50 ms, covering an m/z range of 133.4–2000. For data-dependent MS^2^, the top 10 most abundant precursor ions from each full scan were selected for fragmentation using a 1.0 Da isolation window and a stepped normalized collision energy of 15, 25, and 35 units. MS^2^ spectra were recorded at a resolution of 17,500 with an automatic gain control target of 2 × 10^5^ and a maximum injection time of 100 ms. Lipid identification and data processing were performed using LipidSearch software (version 5.0 SP, Thermo Fisher Scientific) (Taguchi and Ishikawa, 2010).

### Software platform and availability

*LipidCruncher* leverages a comprehensive suite of Python libraries selected for their specific strengths in data science and visualization. For data manipulation and analysis, we rely on *pandas* for its robust DataFrame structure and *numpy* for efficient numerical operations. Statistical analyses were implemented using *scipy.stats* for hypothesis testing and *statsmodels* for multiple comparison corrections with methods, such as the Benjamini-Hochberg procedure. For visualization, *LipidCruncher* used *Plotly* when user interaction with the figure is essential, providing features, such as zooming, hovering, and filtering. We opted for *Matplotlib/Seaborn* when static visualizations were sufficient, with both approaches delivering publication-quality graphics. We incorporated *scikit-learn* to perform PCA. The entire application was built on *Streamlit*, which enables the development of interactive web applications with Python code only. Backend processing included *reportlab* and *svglib* for PDF report generation and *PIL* for image processing. *LipidCruncher* was deployed on Amazon Web Services (AWS) using Elastic Container Service (ECS), ensuring scalable and reliable accessibility for researchers regardless of their computational resources or geographical location.

*LipidCruncher* is available to the research community for free at https://lipidcruncher.org/. The complete source code of LipidCruncher is available on the Farese and Walther Lab GitHub at https://github.com/FareseWaltherLab/LipidCruncher, giving researchers the opportunity to evaluate all analytical choices made during the development of the application.

### Statistical analysis framework

Our statistical analysis framework implements several nuanced approaches for robust lipidomic data evaluation. For error bar calculations on log-transformed data, we apply error propagation principles to accurately represent uncertainty. When transforming from linear to logarithmic scale, the standard deviation in log space is calculated using the formula σlog ≈ σlinear/(mean_linear × ln(base)), where “base” is 10 for log10 and 2 for log2 transformations. This mathematically sound approach ensures appropriate error representation when visualizing data on logarithmic scales, provided the relative error is moderate. The PCA implementation applies *StandardScaler* preprocessing to handle the wide dynamic range of lipid concentrations, followed by confidence ellipse generation using *eigendecomposition* of the covariance matrix. These ellipses represent the 95% confidence region based on a chi-squared distribution with two degrees of freedom, providing visual confirmation of sample clustering reliability. For hierarchical clustering in heatmaps, we employed Ward’s method with Euclidean distance metrics to minimize the variance within clusters, determining optimal cluster numbers through an interactive user selection interface.

Statistical testing functionality automatically selects between Welch’s t-test for two-group comparisons and one-way ANOVA with Tukey’s HSD for multi-group comparisons. When performing log transformation for statistical analysis, *LipidCruncher* imputes missing or zero values using the smallest non-zero value for each lipid class in each sample divided by 10. This imputation strategy assumes that zeros represent values below the detection threshold of the mass spectrometer rather than true absence of the lipid. While we log transform the data to make it more normally distributed, *LipidCruncher* applies statistical tests without explicitly checking normality assumptions. Though Welch’s t-test handles unequal variances, standard ANOVA does assume equal variances, which we do not verify. We recommend interpreting statistical results as exploratory guides rather than definitive conclusions. The automated significance testing provides a systematic approach to identify potentially interesting lipid changes, but researchers should consider these results in the context of biological relevance, magnitude of change, and consistency across related lipid species. We encourage users to verify notable findings with additional experimental approaches and to consider the biological plausibility of results, especially when working with small sample sizes or highly variable measurements.

## Supplementary Material

Supplement 1

## Abbreviations

BQC: batch quality control
CoV: coefficient of variation
DGAT1: diacylglycerol acyltransferase 1
DGAT2: diacylglycerol acyltransferase 2
iWAT: inguinal white adipose tissue
LC-MS/MS: liquid chromatography coupled to mass spectrometry
SFA: saturated fatty acids
MUFA: mono-unsaturated fatty acids
PCA: principal component analysis
PUFA: poly-unsaturated fatty acids
WT: wild type
ADGAT-DKO: Adipose-specific DGAT double knockout
AcCa: acyl carnitine
BMP: bis(monoacylglycero)phosphate
Cer: ceramide
ChE: cholesteryl ester
CL: cardiolipin
Co: coenzyme
DG: diacylglycerol
HexCer: hexosylceramide
LPC: lysophosphatidylcholine
LPE: lysophosphatidylethanolamine
PC: phosphatidylcholine
PE: phosphatidylethanolamine
PG: phosphatidylglycerol
PI: phosphatidylinositol
PS: phosphatidylserine
SM: sphingomyelin
TG: triacylglycerol
