# Supplementary material for: LipidCruncher: An open-source web application for processing, visualizing, and analyzing lipidomic data
Source: bioRxiv. 2025 May 1:2025.04.28.650893. Preprint. [Version 1] doi: 10.1101/2025.04.28.650893 (PMC12247890; doi:10.1101/2025.04.28.650893)
Supplement: Supplement 1 [file media-1.zip › user_guide.pdf]

LipidCruncher User Guide

LipidCruncher is an intuitive and user-friendly web-based application designed for streamlined lipidomics data analysis. Developed with ease of use in mind, its interface is so straightforward that most users may not need a guide. However, this user guide is provided to highlight the most critical user interactions, ensuring a smooth experience for all.

Getting Started

Visit https://lipidcruncher.org/. Scroll to the *Get Started* section and click the *Start Crunching* button. Note: Steps 1–6 are performed in the app's sidebar.

Sidebar Configuration (Steps 1–6)

1. **Select Dataset Format**
   Choose your dataset's format. For this guide, we use the case study dataset (Supplementary File 2) in LipidSearch 5.0 format.
2. **Upload Dataset**
   Upload your dataset file.

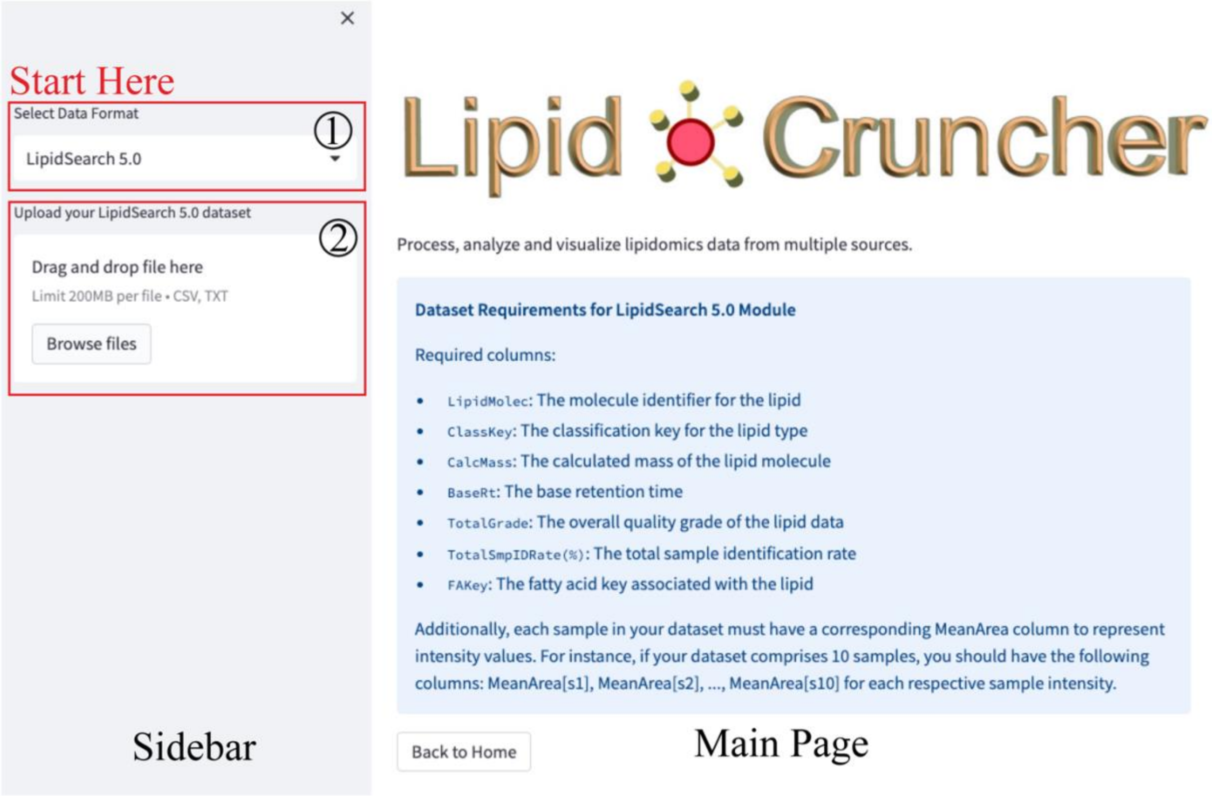

3. **Define Experiment**
   Specify experimental conditions and replicates. For the case study:
   - Conditions: WT, ADGAT-DKO, BQC
   - Replicates: 4 per condition

4. **Verify Sample Grouping**
   Confirm that samples are grouped correctly (e.g., WT samples labeled as s1–s4). If correct, proceed. If incorrect (e.g., WT samples labeled as s1, s2, s11, s12), select *No* and re-arrange the samples. For the case study, the grouping is correct.
5. **Specify BQC Samples**
   Indicate if your dataset includes BQC samples. If yes, provide their label.
6. **Review Experiment Details**
   Review the experiment configuration summary. If accurate, check the confirmation box to proceed to the main app interface. If incorrect, revise the inputs.

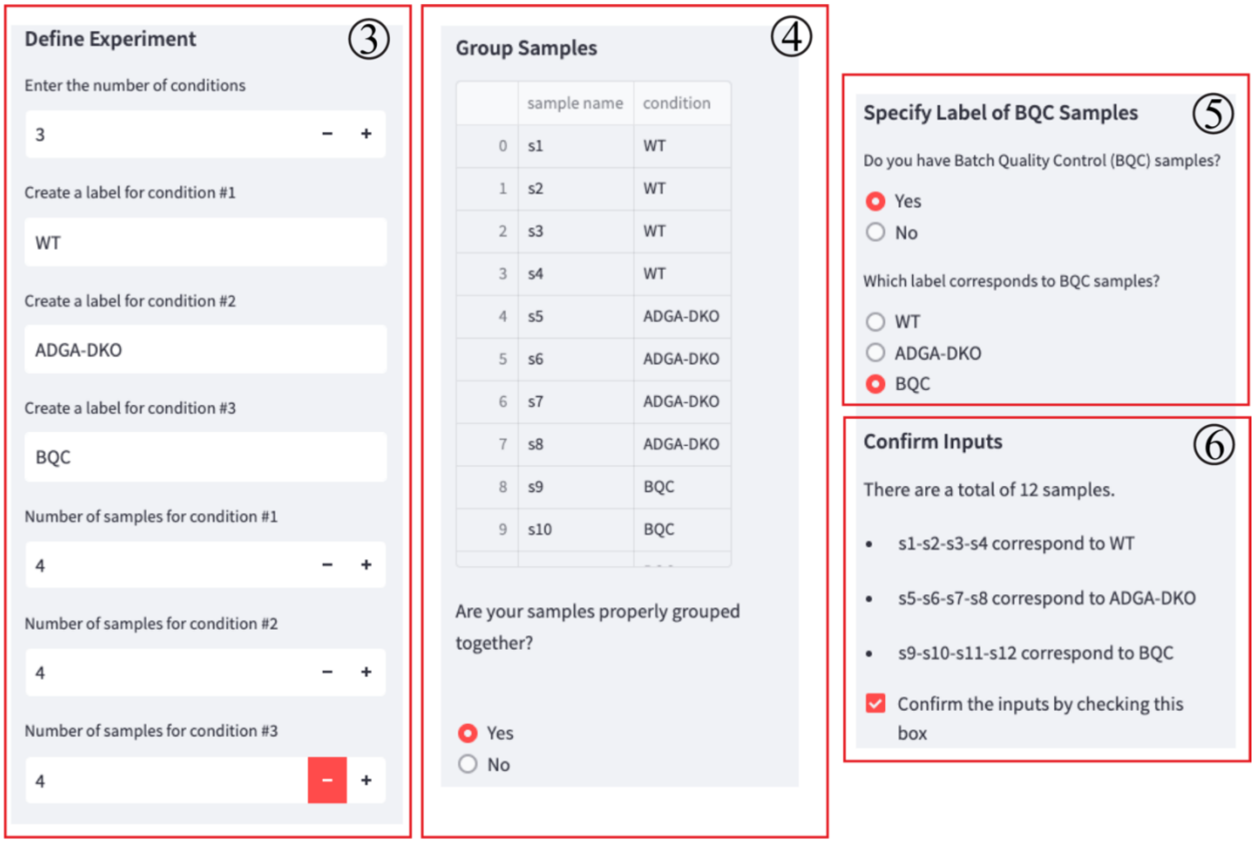

Module 1: Data Standardization, Filtering, and Normalization (Steps 7–11)

Upon confirming sidebar inputs, Module 1 loads automatically, performing data standardization and initial filtering without user input. You can view or download the cleaned dataset. Data normalization, however, requires the following user interactions:

7. **Manage Internal Standards**
   Check the automatically detected internal standards. If they align with your expectations, proceed. If not, select *Upload Custom Standards* and follow the prompts to upload the correct standards.
8. **Select Lipid Classes**
   Choose the lipid classes for analysis.
9. **Choose Normalization Method**
   Select a normalization method:
   - None (select if data is already normalized)
   - Internal Standards

- Protein-Based
- Both

10. **Internal Standards (if selected)**
    Assign an internal standard for each lipid class.
11. **Input Concentrations**
    - For Internal Standards: Enter the concentration for each standard.
    - For Protein-Based: Provide the protein concentration for each sample.
    - For Both: Supply inputs for both methods.

After completing normalization, click *Next: Quality Check & Analysis* to proceed.

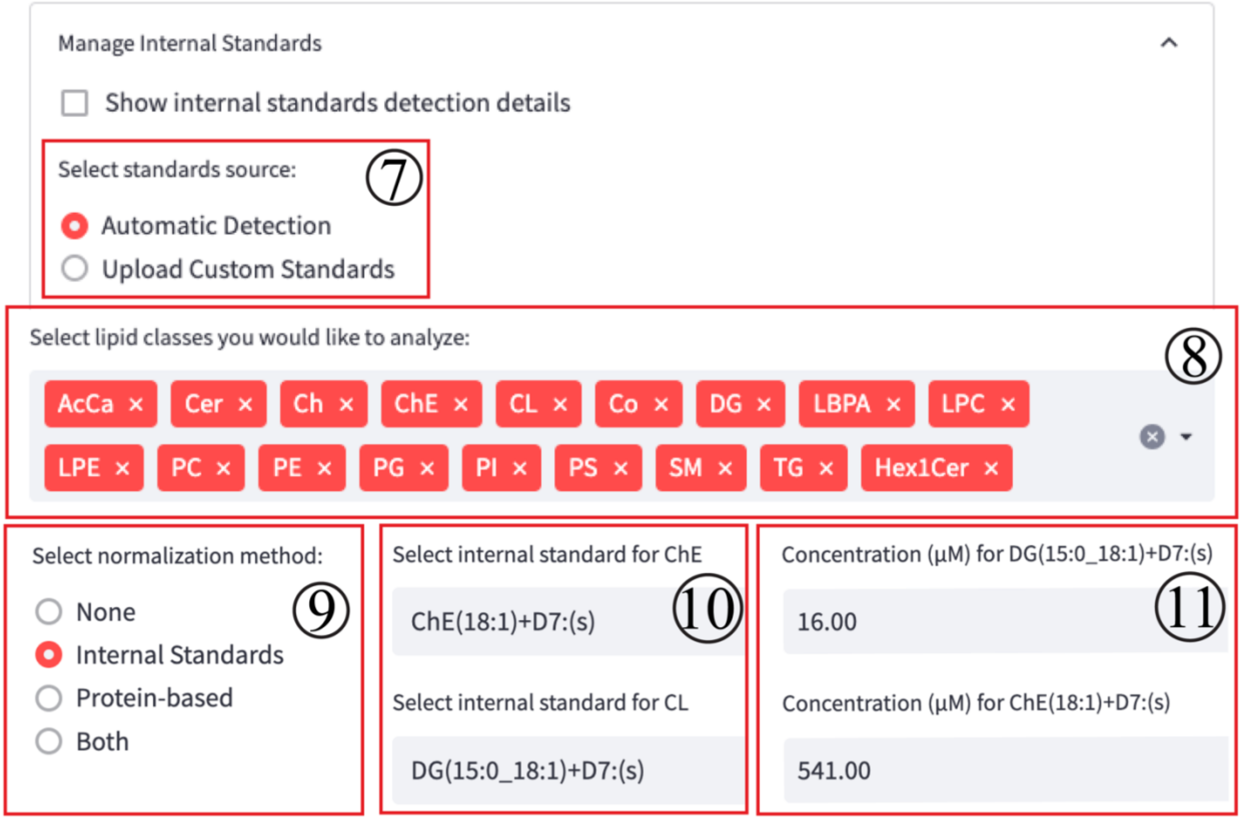

Modules 2 & 3: Quality Check and Analysis (Steps 12–14)

The app now loads the *Quality Check* and *Analysis* modules. Navigation is intuitive, with the following key decision points:

12. **Quality Check with BQC Samples (if applicable)**
    If BQC samples are included, the *Quality Check Using BQC Samples* box appears. Set a Coefficient of Variation (CoV) threshold to filter lipid species exceeding this value.
13. **Principal Component Analysis (PCA)**
    In the PCA box, review sample distribution and remove any anomalous samples from further analysis.
14. **Explore Visualizations and Analyses**
    Select desired visualizations or analyses and explore results interactively.

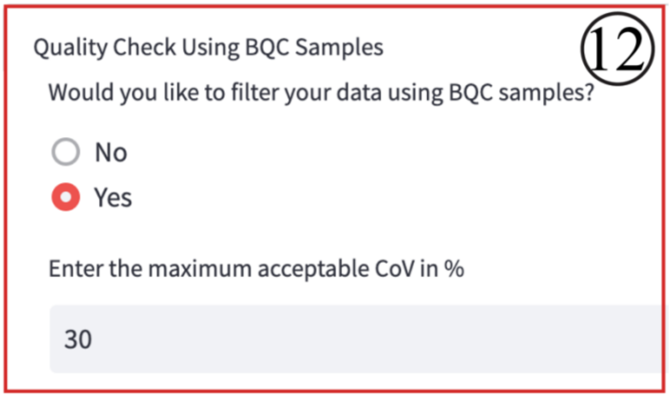

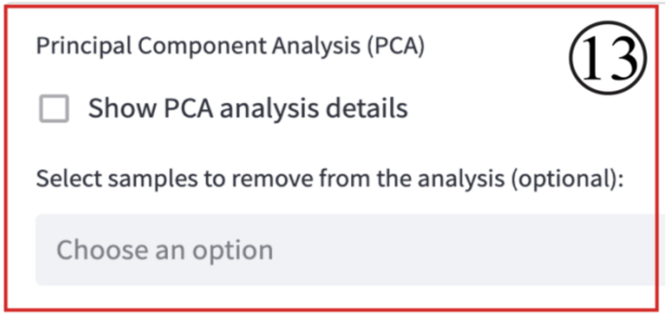

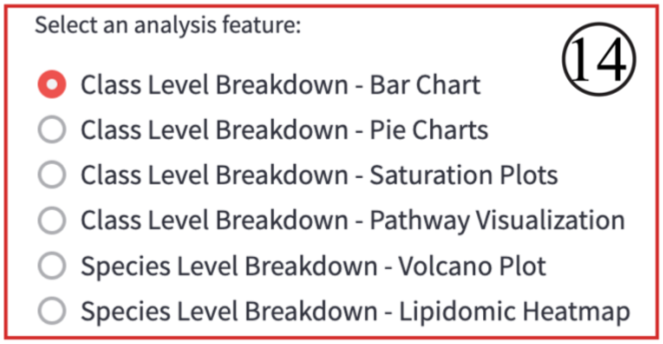

Support

If additional support is needed, email **abdih@mskcc.org**.

Happy crunching!
